# Complete Digital Workflow for Manufacturing Presurgical Orthodontic Palatal Plates in Newborns and Infants with Cleft Lip and/or Palate

**DOI:** 10.3390/jfb15100301

**Published:** 2024-10-08

**Authors:** Christina Weismann, Alexander B. Xepapadeas, Marit Bockstedte, Bernd Koos, Michael Krimmel, Christian F. Poets, Maite Aretxabaleta

**Affiliations:** 1 Department of Orthodontics, University Hospital Tübingen, Osianderstr, 2-8, 72076 Tübingen, Germany; christina.weismann@med.uni-tuebingen.de (C.W.); alexander.xepapadeas@med.uni-tuebingen.de (A.B.X.); marit.bockstedte@med.uni-tuebingen.de (M.B.); bernd.koos@med.uni-tuebingen.de (B.K.); 2 Centre for Cleft Lip, Palate and Craniofacial Malformations, University Hospital Tübingen, Osianderstr, 2-8, 72076 Tübingen, Germany; michael.krimmel@med.uni-tuebingen.de (M.K.); christian-f.poets@med.uni-tuebingen.de (C.F.P.); 3 Department of Oral and Maxillofacial Surgery, University Hospital Tübingen, Osianderstr, 2-8, 72076 Tübingen, Germany; 4 Department of Neonatology, University Hospital Tübingen, Calwerstr, 7, 72076 Tübingen, Germany

**Keywords:** unilateral cleft lip and palate, bilateral cleft lip and palate, cleft covering plate, computer-aided design, computer-aided manufacturing, 3D printing, intraoral scanning

## Abstract

Deciding on the implementation or modification of steps in daily clinical care is a nuanced process that demands careful evaluation. This is crucial not only for selecting the most appropriate solution but also for achieving the best treatment outcome. Thus, implementing a workflow for treating cleft lip and/or palate patients with a presurgical orthodontic cleft-covering plate needs to consider objective factors, prioritized from most to least important: safety and quality level, user-friendliness, feasibility, and, finally, efficiency and cost. The goal of this workflow is to integrate CAD/CAM technologies into daily clinical routine to enhance technical and clinical efficiency, reduce the burden of cleft care, and simplify the implementation of these technologies in other facilities. To achieve this, a methodology based on intraoral scanning and additive manufacturing is employed to produce patient-specific passive palatal plates. The approach describes possible pitfalls and their resolution within the routine of a cleft centre, along with an exemplary case scenario. Comparative analysis between the digital workflow and the conventional process demonstrated the digital approach to be safer, higher in quality, more user-friendly, feasible, and cost- and time-effective than the conventional process.

## 1. Introduction

Cleft lip and/or palate (CL/P) is the most common craniofacial disorder (CD), affecting about 1:600 live births [1]. The anatomy of the cleft varies in its width and collapsibility of the maxillary lateral segments. Wider clefts are associated with an increased nasal deformity and tissue deficit, particularly in the palatal region. This decreases both aesthetics and oral function, making rehabilitation challenging; thus, an early start of treatment is desirable. This can be achieved by using a patient-individual presurgical orthodontic plate that covers the palate starting at birth. These medical appliances not only aim to physically separate the oral from the nasal cavity to enable a physiological tongue position, but also prevent the tongue from entering the cleft. Hence, feeding difficulties can be reduced [2]. These appliances, also known as Presurgical Infant Orthopaedics (PSIO), guide maxillary growth and reduce disturbing factors. Treatment should take advantage of the growth of the alveolar ridges and result in a physiological arch form [2]. In addition, a smaller cleft width or closer alignment of the segments may facilitate the subsequent surgical closure, as the defect can be bridged with less tension to the tissue. Moreover, it decreases post-operative complications and scarring, which might act as inhibitors to maxillary growth [3].

In 1950, McNeil was the first to use orthodontic palatal plates for arch alignment [4], where he adapted the following theory by the anatomist Scott: “The growth deficiency of the maxillary complex is due to the palatal segments were detached from the forward-downward growth of the nasal complex, the growth centre of the midface” [5]. Based on his studies, many clinicians included early presurgical palatal plates in their treatment concept [6,7,8,9,10,11]. Among the proposed approaches, three distinct methods can be identified: passive plates [4,6,12,13,14], semi-active devices with screws and springs [15,16], as well as their combination with extraoral lip taping [17,18], and active pinned retractive appliances [19]. The evolution of palatal plate devices continued, with a significant development occurring in 1993, when Grayson et al. added an extraoral nasal stent to the palatal plate to mould the nasal cartilage, called nasoalveolar moulding (NAM) [20]. This approach aimed at improving the aesthetics of the nose, while retaining the positive effects of the cleft covering plate [21].

Among the most famous approaches, the Zurich Concept, developed by Hotz and Gnoinski in 1960, should be emphasised [12,13,14]. Their so-called Zurich plate consists of a passive device, created with soft and hard acrylic, which extends into the nasal cavity and has a posterior extension creating an uvula to facilitate the swallowing process [12]. The appliance is indicated for the first 16–18 postnatal months and requires adjustments every 3–8 weeks. Here, selective grinding in the region of the alveolar segments is necessary to provide space for its growth guidance [12]. While this concept focuses on directing growth of the alveolar segments, it does not aim to stimulate growth [12,13,14], as other authors assert for their respective approaches [4,11]. The Zurich concept involves lip closure at 6 months of age, soft palate closure at 18 months, and finally closure of the hard palate at around 4–5 years [12,13]. It adheres to the principles of creating space in areas deficient in tissue with the aim of shaping a physiologically appropriate dental arch. This process requires the orthodontist to possess expertise, as well as comprehensive understanding and careful consideration of the anatomical nuances of maxillary shape and growth. Moreover, the palatal plate needs to be replaced every 3–4 months, depending on the patient’s growth. Furthermore, extraoral taping alongside the palatal plate is performed with the goal of repositioning the flared cleft segments into a physiological position [22]. While the Tübingen approach follows the same concept, with lip closure performed at age 6 months, it differs in its surgical approach, as both the soft and hard palate are closed in a single surgical procedure at age 1–1.5 years. After the initial surgery for lip closure, a new palatal plate must be manufactured to account for the emerging lip tension.

Implementation of digital technologies can aid addressing some of the drawbacks associated with conventional approaches, especially in treatments that rely on personalized medical devices. This approach proves most beneficial for rare diseases where mass production it not feasible. Patient individualization is a key element here. Due to substantial technological advancements in recent years, CAD/CAM (Computer-Aided Design and Computer-Aided Manufacturing) technologies are now firmly established within the market for personalized devices [23,24,25]. Various CAD software tools enable the design and production of patient-specific devices through additive (AM) or subtractive manufacturing (SM). Their implementation has the potential to facilitate a routine in-house approach for treating infants with CL/P through presurgical palatal plates. Given that regular adjustments and new appliances are essential due to physiological growth, a labour-intensive clinical workflow becomes imperative. Historically, this started with alginate impression-taking, a procedure that posed risks [26]. Impression material could tear off, remain unnoticed and initiate an inflammatory response by the surrounding tissue. In the worst case, it could get aspirated [27,28]. Thus, to avoid such potentially life-threatening situations, securing the upper airway through intubation and sedation was necessary to avoid cyanotic events [26,29]. Here, intraoral scanning (IOS), introduced by our group for infants with CL/P, Trisomy 21 and Robin sequence, has become the standard procedure in our centre [30,31]. We have described digital workflows for such applications, utilizing CAD/CAM tools for the design and manufacturing of stimulation plates [32] and Tübingen palatal plates (TPPs) [25,33,34,35]. There are yet only few approaches in a limited number of patients [36,37,38], but at our centre, it has made the conventional impression taking and manufacturing obsolete. This happened after careful consideration of several factors, organized from most to least important: Safety level, quality level, user-friendliness, feasibility, efficiency, and cost. By now, every CL/P patient is treated employing exclusively IOS and CAM, following a methodology that is intended to be introduced within this study. 

The primary aim of this report is to introduce our CAD/CAM-based in-house palatal plate workflow and demonstrate its integration into the interdisciplinary Tübingen approach. This implementation should enhance technical and clinical efficiency, reduce the burden of cleft care, and aid in the implementation of such technologies in other facilities. To illustrate its feasibility, we showcase a patient case treated using this methodology within the interdisciplinary approach.

## 2. Materials and Methods

This manuscript consists of three parts: a technical section detailing each step of the CAD/CAM-based workflow, a clinical section describing its implementation in the interdisciplinary treatment, and a section providing practical advice. A detailed case of a non-syndromic right-sided cleft lip and palate (CLP) patient is presented to illustrate the workflow and clinical application. Practical advice, along with time and cost efficiency considerations, is provided to support adoption by other cleft centres and ease its implementation.

**Figure 1:**
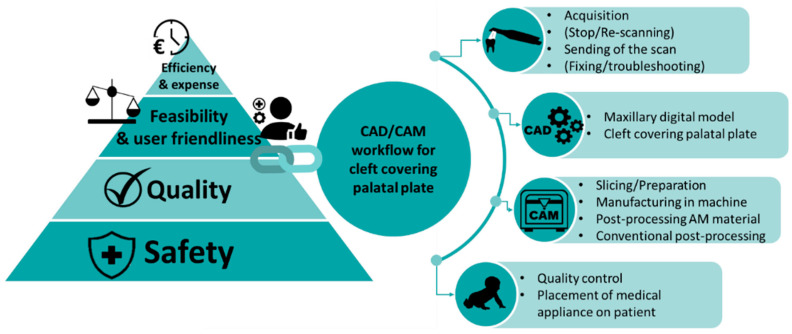
Overview of the steps involved in a CAD/CAM workflow for cleft covering palatal plates, along with the essential criteria for ensuring its successful implementation in a hospital setting (Steps within brackets indicate optional procedures).

The complete digital workflow for the creation of patient-individualized palatal plates encompasses four primary steps (Figure 1). First, patient-specific information is acquired through IOS to obtain 3D image data of the patient’s maxilla. Next, these data are processed using CAD tools to digitally design the maxillary model and personalized palatal plate. These are manufactured using CAM technologies and post-processed. Lastly, quality checks and plate fitting evaluations are performed. Based on previous studies on IOS protocol [30], CAD/CAM fabrication of palatal plates for other CDs [32,33] and in vitro CAM material tests [31], the presented workflow has been employed and optimized since 2018 to address various scenarios and challenges.

The study was conducted in accordance with the Declaration of Helsinki and approved by the Institutional Review Board (or Ethics Committee) of Tuebingen University hospital (protocol code 455/2019BO2 and date of approval 13 August 2019).

### 2.1. Patient Data Acquisition through Intraoral Scanning

**Figure 2:**
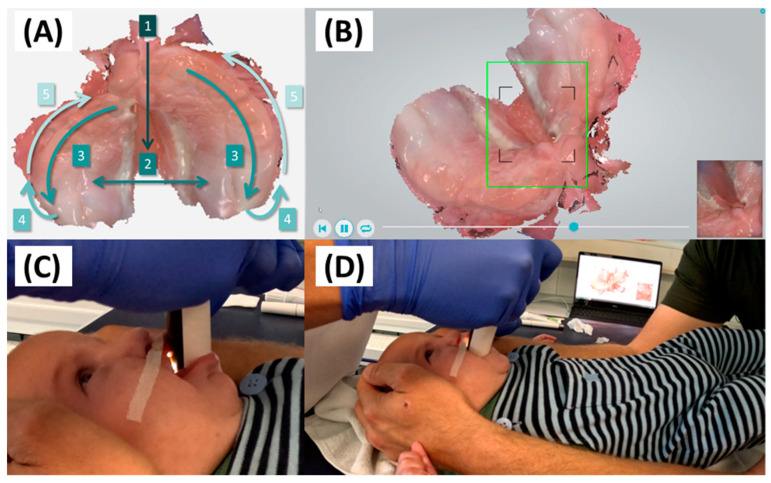
Scan protocol for a 2.5-month-old patient with right-sided CLP (corrected age 2 months) followed the published protocol [30]. The patient was placed in a supine position for easy mouth access, with data monitored on the scanner’s laptop. (**A**) Sequential scanning steps respectively (1-5): incisive papilla, palatal area, alveolar ridge, posterior, and anterior muco-buccal vestibulum. (**B**) Data synchronized with real images (bottom right corner) enabled clinician oversight. (**C**) Close-up image of the scan head in the patient’s mouth. (**D**) Proper clinician, laptop, and patient positioning, with parental help to restrict movement. [Parental informed consent was obtained to publish the information/image (s) in an online open access publication].

IOS serves as the start for the digital workflow, utilizing confocal scanners from 3Shape, eligible for both Trios 3 and Trios 4 (3Shape A/S, Copenhagen, Denmark). These scanners have proven clinically feasible in capturing images of newborns and patients with CD, despite differences in built-in technology and scanner head size [30]. Weekly calibration, per manufacturer guidelines, ensures precise image acquisition and texture quality. The software automatically records the duration and number of images captured during IOS. Scanning takes place chairside during routine orthodontic visits, without sedation or fasting. To capture crucial anatomical structures, the procedure previously developed by Weise et al. [30] is consistently applied (Figure 2).

The patient’s clinical condition and cooperation can significantly impact the obtained scan quality, particularly in challenging anatomical conditions like a large cleft. Operator training level, software issues, and PC performance, also contribute to challenges. Despite the scanner software’s provision of intelligent and automatic artefact removal, situations arise where avoiding scanner-generated artefacts proves impossible. Artefacts are defined as any image or registered mesh region that does not accurately represent the patient’s actual maxilla.

**Figure 3:**
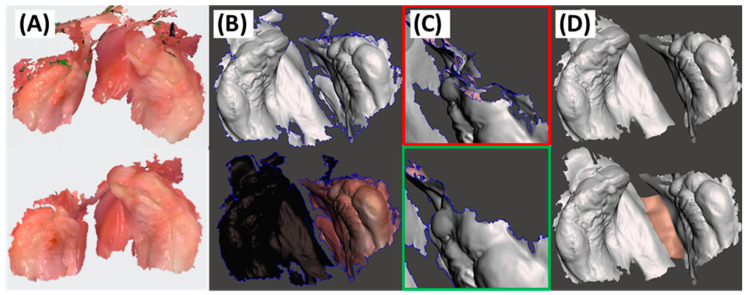
Different methods for addressing scan artefacts are demonstrated in two distinct scan sets using different software (**A**–**D**). (**A**) Artefact removal through both automatic and manual steps in the 3Shape scanner software, depicted before (**top**) and after (**bottom**). This involves the automatic detection of major artefacts not connected to the main structure, with certain holes covered using patches (green). The bottom image displays the outcome after utilizing the “cut” function. (**B**) Identification of unconnected segments in Meshmixer, with the unconnected segment lacking colour and texture information. (**C**) Manual artefact removal by Meshmixer before (red) and after (green). (**D**) Elimination of parts between major and minor segments, followed by smoothing corners before creating an artificial connection (“Bridge” function).

These artefacts are typically separate from the main scan file (Figure 3). The initial option is manual removal using the “cut” function in the scanner software, enabling deletion of user-selected areas from the primary scanner region. If these areas are within a critical scan zone, they can be eliminated and the area re-scanned. While minor artefacts are generally insignificant and easily addressed in subsequent software processing, challenging clinical and anatomical conditions may hinder desired outcomes even with post-processing or re-scanning efforts. In such cases, mesh editing freeware software like Meshmixer V3.5.474 (Autodesk Inc., San Rafael, CA, USA) becomes valuable, allowing for the selection and removal of artefacts (Figure 3B,D).

Complications may particularly arise in larger or wider cleft defects. When the original software processes both maxillary segments as separate entities, additional software tools become necessary. In such cases, one side is categorized as a large artefact, complicating further CAD step calculations. For example, Weise et al. [30] suggested to create a “virtual bridge” between segments using a cotton swab or a wound compress, but maintaining such items securely and avoiding movements during scanning poses challenges, especially in a highly mobile patient. Thus, it is preferable to perform scans without such tools and to artificially create a connection between the segments later using other software, such as the process in Meshmixer shown in Figure 3B–D. 

### 2.2. Computer-Aided Design (CAD) of the Dental Maxillary Model

After the scan, commercially available software (3Shape A/S) is employed for maxillary model creation and subsequent plate design. Scan information is automatically sent between programs. When manual repair of the scan is required, it can be uploaded in both .stl (Stereolithography file format) and .ply (Polygon file format) formats, with the latter containing color and texture information. 

**Figure 4:**
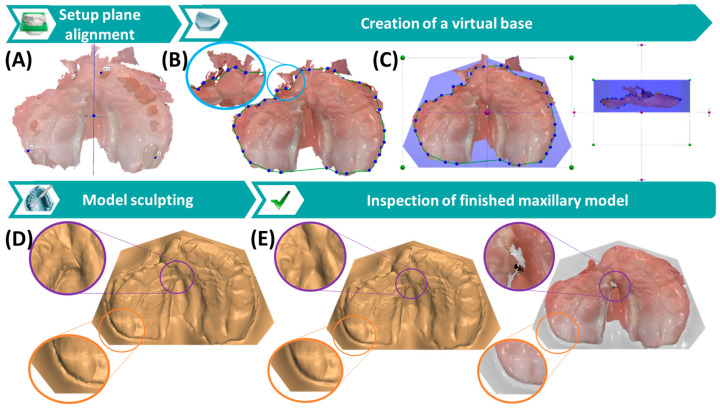
Intraoral scan-based creation of a maxillary digital dental model using CAD solutions and involving four main software modules: Setup of plane alignment (**A**), creation of a visual base (**B**,**C**), model sculpting (**D**), and inspection of the maxillary model (**E**). (**A**) Alignment in respect to the occlusal and sagittal planes of the scan. (**B**) Selection of the area of interest through a spline with a close-up image of the removed region. (**C**) Adjustment of socket (blue box) orientation and dimensions using different view modes (i.e., top view left, lateral view right). (**D**) Result after the automatic socket creation with close-up images depicting artefact creation, particularly at the scan-socket base connection. (**E**) Result of the sculpting procedure, with its effect visible in both textured (**right**) and non-textured (**left**) models. Areas with fixed major artefacts are displayed in black.

For maxillary model creation, the 3Shape Appliance Designer software (3Shape A/S) is utilized, featuring four main modules or windows (Figure 4): setup plane alignment module, creation of virtual base, model sculpting, and inspection of finished maxillary model. To align the scan with the occlusal plane, areas corresponding to teeth 11–21 in the premaxilla and teeth 16 and 26 of the posterior region are selected. The sagittal plane is chosen at the approximate middle width of the maxillary scan (Figure 4A). Next, areas of interest are selected using a spline, excluding artefact regions or unnecessary details (Figure 4B). Subsequently, the virtual base is edited and optimized to encompass the scan and its corresponding spline, considering different viewpoints for minimal size while retaining patient information (Figure 4C). Once the base shape is adjusted, the program automatically creates the model. Artefacts may arise at the borders between the scan and socket, necessitating removal using software tools in the model sculpting module (Figure 4D,E). At each step, the option to remove the scan’s colour texture exists, aiding in structure and error identification, especially during model inspection. Coloured regions may indicate previously fixed major defects, which should be considered in subsequent steps.

### 2.3. Computer-Aided Design (CAD) of the Presurgical Orthodontic Palatal Plate

**Figure 5:**
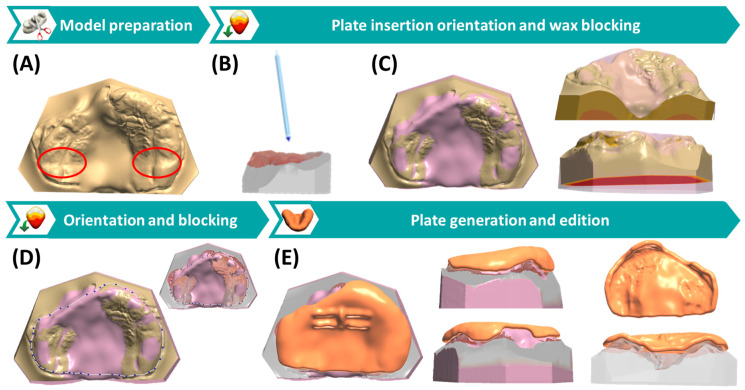
Digital maxillary model-based CAD design and creation of the cleft covering plate, involving three main software modules: Model preparation (**A**), plate insertion orientation and wax blocking (**B**–**D**), plate generation and freeforming (**E**). (**A**) Model preparation, where deep regions of the model are edited. Red circles mark the indentation of the previous palatal plate. (**B**) Simulation of the orientation of palatal plate placement. (**C**) Blocking of regions by digital wax, where not only the cleft but also the previous mark indentations are blocked. (**D**) Definition of the contour of the palatal plate through a spline in the model-wax setup, while controlling the outline by the texture scan model. (**E**) Final palatal plate after automatic generation and finishing steps in different views.

For the creation and design of the patient-specific appliance, 3Shape Dental software (3Shape A/S 2023, v.1.11.2.0.) is employed. Here, the setup for creating individual tray impressions is utilized, and the previously designed maxillary model is imported. The program comprises three distinct modules or viewports: model preparation, plate insertion orientation and wax blocking, as well as plate generation and edition (Figure 5). Initially, the original maxillary model is adapted, involving the editing or elevation of deep cleft regions for subsequent blocking (Figure 5A). Careful adjustment is essential, as only a limited amount of digital wax material is allowed to be placed later. Subsequently, the orientation for plate insertion is defined (Figure 5B), serving as a basis for the computer to automatically block regions affecting appliance placement, such as model undercut structures and sharp areas. Wax material is added to block hard and soft regions, including the alveolar cleft. No modifications are made to other anatomical structures, as these may impair the fit of the appliance.

As previously mentioned, the Tübingen approach employs a passive palatal plate, following the concept of Hotz and Gnoinski [12,13,14]. This method employs digital wax to cover the cleft in both, hard and soft palatal regions, including the alveolar ridge (Figure 5C,D). By simulating a physiological dental arch without cleft using wax, space is generated for lateral cleft segments, guiding their orthopaedic growth. The objective is not only to reduce the cleft extension, but also to address vertical and sagittal deficits commonly associated with such cases, aiming for well-aligned maxillary segments. In the lesser and collapsed segment, wax is added in the vestibular and anterior area to encourage growth in this direction. Close collaboration between technician and clinician in the latter step is paramount for ensuring treatment success.

Additionally, other areas require blocking to ensure healing or normalization of the injured mucosa, falling into two categories based on their origin. First, pressure marks may arise already in utero, with the tongue exerting pressure on the delicate nasal mucosa, especially at the vomer. Second, mucosal indentations may occur due to delayed plate renewal or rapid patient growth. In such instances, the pressure of the tongue against the palatal plate leads to rim formation in areas of misaligned or poorly fitting areas, attributed to the resilience of the gingiva. Posterior indentations of the maxilla are most typical (Figure 5A). Both mucosal peculiarities, however, may fully heal without consequences by providing space between palatal plate and mucosa. This can be accomplished by blocking these areas with digital wax (Figure 5C). 

Following wax blocking, a spline defining the shape or contour of the palatal plate is defined on the model with wax (Figure 5D). Median and lateral lip ligaments are spared to prevent injuries and ensure their correct physiological movement. While median ligaments are easily recognizable, identifying structures of the lateral ligaments can be more challenging, and removing colour and texture can assist in this process. If this is not possible, the necessary space can be produced by grinding down the region chairside. Additionally, the spline should extend deep into the vestibulum and as posterior as possible to ensure a secure fit of the appliance. In the alveolar cleft region, the spline follows the most elevated area of the digital wax, mimicking the physiological shape of the alveolar ridge (Figure 5D). In cases of clefts with alveolus and lip cleft, the spline is not set at the base of the vestibulum to guarantee enough space for lip taping and movement. Through extraoral taping and subsequent lip surgery, the goal is to guide this area without the need for plate assistance. A model with texture (Figure 5D) is used to verify that the selected regions for the plate calculations correspond to the scanned structures and do not originate from the base. Additionally, this texture aids in identifying model artefacts created beforehand, requiring precautionary blocking. This process ensures that the final plate is not created on artificial data, reducing potential fitting problems or pressure marks on the gingiva. 

Once the spline is set, the program automatically generates the plate by extruding the splint shape to a minimum thickness of 2 mm (Figure 5E). The occlusal side of the plate is smoothed to achieve a homogeneous occlusal plane, ensuring no interference with a correct bite. Finally, corners and inside areas of the plate are inspected for possible minor artefacts, which are smoothed using the smallest altering tool (µm level). Lines simulating the palatal rugae can be created on the surface to create a physiological feeling for the tongue and improve the usual tongue resting position. However, if these lines cause produce discomfort, they can easily be polished down.

### 2.4. Computer-Aided Manufacturing (CAM) of the Presurgical Orthodontic Palatal Plate

**Figure 6:**
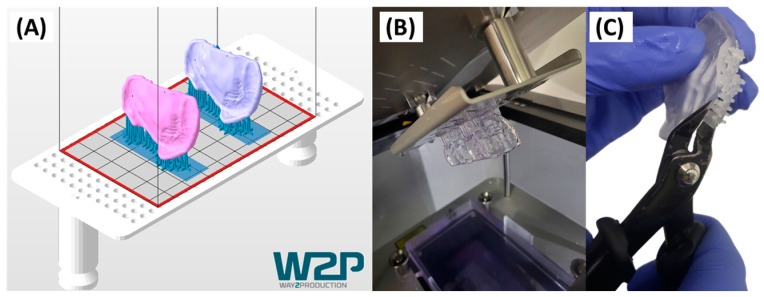
Additive manufacturing of the patient-individualized cleft covering palatal plate. (**A**) Orientation, placement and supporting structures of two equal patient plates in Netfabb 2021, employing Solflex 170 platform and VOCO Splint. (**B**) Outcome after manufacturing without post-processing. (**C**) Removal of support structures from the final appliance.

To manufacture the plates, a previous study found that employing Additive Manufacturing (AM), specifically Direct Light Processing (DLP), was the most effective solution [31]. The orthodontic appliances are manufactured from MDR class IIa approved splint material (V-Print Splint Clear, VOCO GmbH & Co. KG, Cuxhaven, Germany) using a DLP machine (Solflex 170, Way2Production, Vienna, Austria) [31,32]. Netfabb Premium 2021 (Autodesk, Mill Valley, CA, USA) is employed for job file preparation. Appliances are positioned to eliminate the need for support structures on the mucosal side of the plate, not only to avoid possible defects caused by their placement and removal but also to increase accuracy of the produced part [31]. The premaxillary region of the plate is oriented towards the platform. This placement is intended for subsequent grinding on the occlusal side of the plate, which removes defects created by support removal. The appliances are raised 2 mm above the platform, and support structures from VOCO are employed. Ensuring an adequate amount of support structures is crucial; however, an excessive amount may risk breaking off during post-processing. Two identical appliances are routinely manufactured for the same patient in a single production run, which ensures fast substitution in case of manufacturing errors, breakage or loss. An example for a final job file is shown in Figure 6A. Following recommendations from a previous study [31], 100 µm layer height is routinely employed to provide the highest part accuracy within the shortest manufacturing time. 

### 2.5. Post-Processing of the Manufactured Appliance

After manufacturing (Figure 6B), parts undergo two different post-processing steps, one based on the specifics of the AM material, the other following conventional dental technical steps. For the former, different washing and curing steps are required. Washing involves a two-step ultrasound cleaning with 98% Isopropanol (IPA): 3 min for the first wash and 2 min for the second. Compressed air is then used to dry and remove any remaining dust or resin. Next, support structures are removed using Finish Kit (Formlabs, Somerville, MA, USA; Figure 6C). For post-curing, a two-step curing process by Otoflash G171 (λ = 280–700 nm, NK-Optik, Baierbrunn, Germany) is performed, with each step consisting of 2000 flashes.

**Figure 7:**
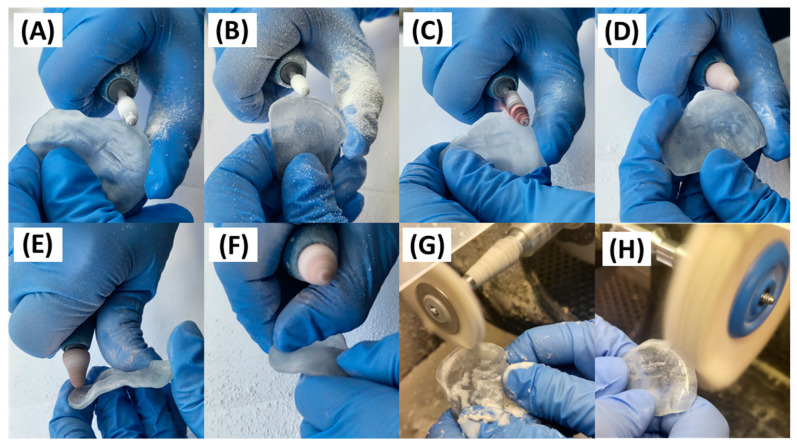
Manual post-processing steps for the cleft covering palatal plate after additive manufacturing. (**A**) Removal of marks from the support structures and the stair-case effect of the manufacturing process using a cutting tool. (**B**) Thinning of the vestibular sides. (**C**) First polishing step using sandpaper. (**D**) Second polishing step by a smaller grain sandpaper tool. (**E**) Smoothening of possible sharp edges in the vestibular inner area of the plate. (**F**) Testing of sharp edges by finger. (**G**) Third polishing step by means of a buffing wheel and pumice powder. (**H**) Final polishing step using buffing wheel and polishing paste.

Finishing steps are performed in the dental lab (Figure 7). Initially, regions with marks from the support structures are ground using a cutter (coarse and bullet cross tooth cut, REF 123-584-00, Dentaurum, Ispringen, Germany), while the vestibular walls of the plate are thinned to approximately 1.3 mm. Subsequently, a two-step polishing process is applied, using sandpaper with decreasing grain sizes. Using the lowest grain sandpaper tool available, sharp edges in the vestibular inner area of the plate are smoothed. Next, mechanical polishing with powdered pumice and polishing with a soft buffing wheel and polishing paste are performed. All manual processing steps described above only concern the lingual side, leaving the mucosal side untouched to ensure the fit of the device, in contrast to the procedure done for prosthetic dentures [31].

### 2.6. Quality Control of the Manufactured Appliance

**Figure 8:**
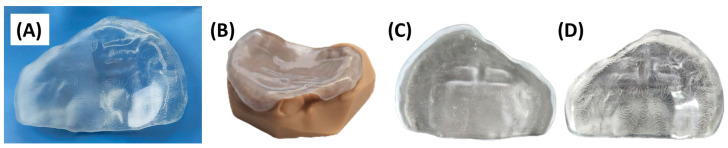
Final cleft covering palatal plate. (**A**) Difference between polished (right half) and unpolished (left half) lingual surface. (**B**) Palatal plate fitting on the model. (**C**) Inner area of the palatal plate is left unpolished for an increased retention. (**D**) Outer area of the palatal plate must be fully polished to ensure no mechanical irritation of the mucosa and to decrease adhesion of bacteria (visible lines correspond to the inner area, shown due to the part´s transparency).

Before using the appliance in the patient, performing a quality control check is essential. Regarding the CAD process, this should ensure maintaining a homogeneous occlusal plane and proper blocking of areas such as prior injuries or indentations. Concerning manufacturing, adherence to MDR guidelines is crucial. Additionally, there should be no defects originating from the manufacturing process, such as microcracks or bubbles. For the conventional steps (Figure 8), thorough polishing of the bottom of the part is imperative to remove any trace of roughness from both the stair-case effect and the polishing steps (Figure 8). This ensures greater comfort for the patient and facilitates cleaning. In the mucosal area of the plate, only pressure areas or sharp edges can be sanded down, as roughness and accuracy in these areas play a vital role in securing the appliance to the maxilla.

### 2.7. Implementation of the Digital Workflow for Bilateral Cleft Cases

**Figure 9:**
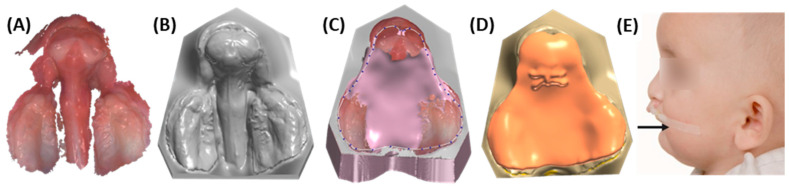
Workflow for bilateral cleft with an extensive cleft defect and maxillary protrusion. (**A**) Intraoral scan. (**B**) Maxillary model without texture. (**C**) Maxillary model with the added wax. An additional layer of wax is added to allow the premaxillary segment to move towards the alveolar segments. (**D**) Maxillary model with generated patient specific appliance. (**E**) A patient with bilateral cleft lip and palate presents with a dislocated, protruding premaxillary segment, which is being stabilized and retracted using lip taping (indicated by the arrow). [Parental informed consent was obtained to publish the information/image(s) in an online open access publication].

The proposed workflow is based on a unilateral CL/P scenario but can also be employed for challenging anatomical conditions, like bilateral cleft cases. The primary difference lies in blocking both alveolar regions using digital wax. For extensive cleft defects, in which the premaxilla protrudes forward, it is not surrounded by the appliance outline. Instead, an additional layer of wax is added in the premaxillary region (Figure 9). This enables movement of the premaxillary towards the space between the lateral segments. This is further aided by the pressure exerted by the lip, either through taping (Figure 9E) or after surgical lip closure.

### 2.8. Implementation of the Digital Workflow in the Interdisciplinary Approach

**Figure 10:**
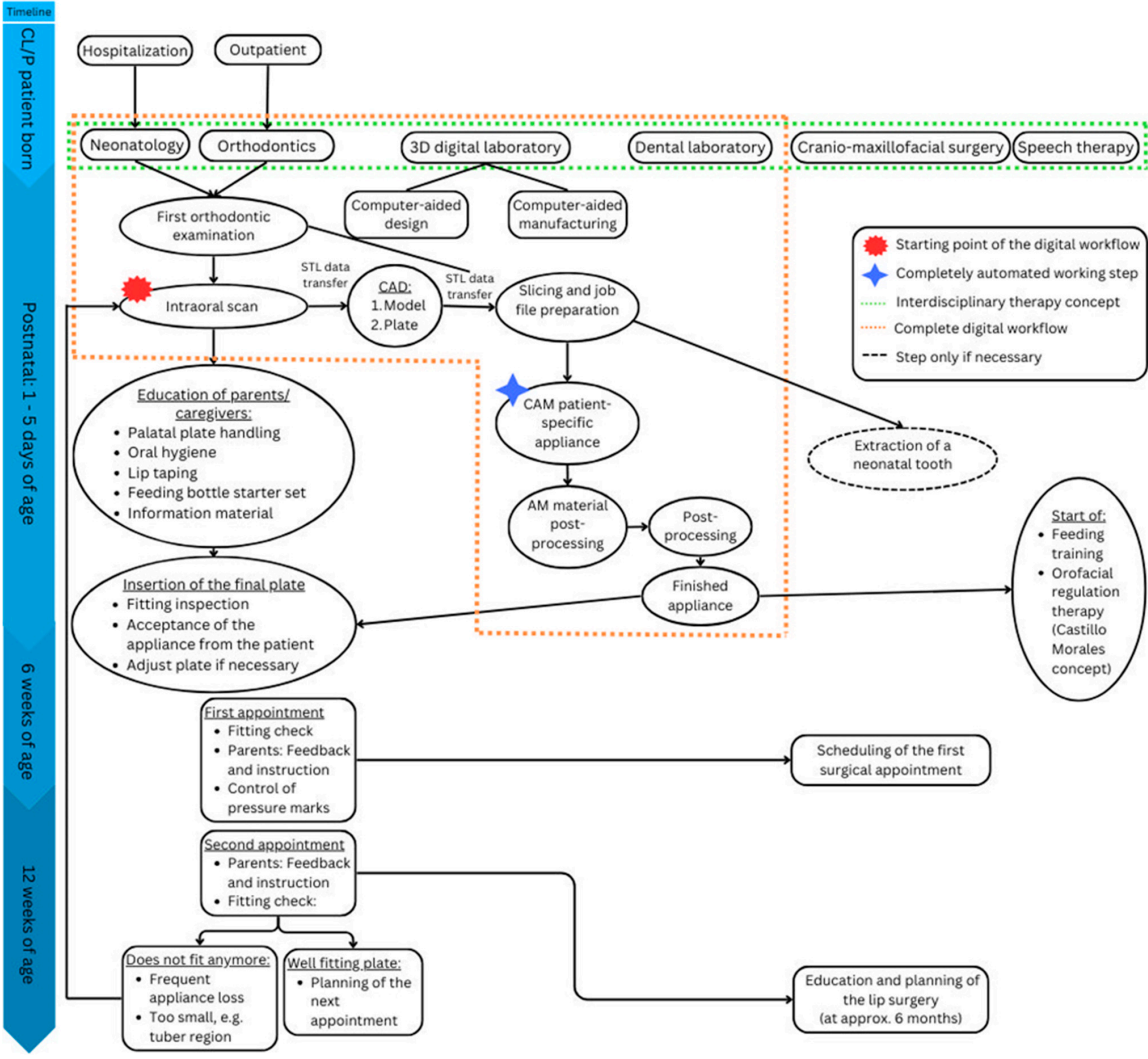
Flowchart showing the digital workflow for manufacturing presurgical orthodontic palatal plates (orange) in the interdisciplinary Tübingen approach (green) for newborns with cleft lip and palate in the first three months of life.

The presented workflow shows that it is possible to manufacture a presurgical orthodontic passive palatal plate completely digitally. Figure 10 displays a detailed flow-chart of the clinical Tübingen approach of cleft care in newborns implementing the digital workflow using CAD/CAM technology. Each step is shown according to the different medical disciplines involved during the first 3 months of life.

## 3. Implementation in the Clinical Routine—Exemplary Case

The presented patient is a newborn boy with a non-syndromic right-sided CLP, who was routinely treated using the above digital workflow (Figure 10). The cleft was known prenatally, and the patient was born in-house at 37 weeks gestational age. On the day of birth, he was introduced to the orthodontists at the Department of Neonatology, where a scan was performed using Trios 4 (3Shape). Afterwards, the palatal plate was designed and manufactured according to the above digital approach. The patient was born at 6 a.m., underwent an intraoral scan at 9 a.m. and had a plate fitting at 4 p.m. (Table 1).

The appliance was inserted and its fit in the oral cavity inspected, following the guidelines summarized in Table 2. No chairside modification was found necessary. A small amount of dental adhesive cream (blend-a-dent, Procter & Gamble Service GmbH, Schwalbach, Germany) was applied to the alveolar ridge. During the same appointment, parents were instructed in handling the plate, everyday oral hygiene, lip taping, and that the plate should only be removed once or twice daily for inspection of the mouth for pressure marks or traumatic ulcerations and cleaning using gauze swabs and Dexpanthenol solution (Bepanthen^®^, Bayer, Leverkusen, Germany) to remove adhesive cream and leftover milk using a tooth brush and tooth paste under running water.

The purpose of lip taping is to bring the malpositioned cleft segments into a physiological position. Surgical tape (Steri-Strip, 3M Health Care, Saint Paul, MN, USA) is connected with an orthodontic elastic band (Intra-oral, non-latex elastics, medium pull, 1.3 N, 1/8 inch, Dentaurum) to provide tension on the perioral muscles and the alveolar bone. This aims to reduce the width of the lip and alveolar gap. Parents receive written information about the Tübingen approach, and a bottle-feeding starter set (Playtex™ Baby, Playtex Inc., Shelton, CT, USA). This bottle system contains a soft bag (so-called “drop-in liner”), a threaded tube, a teat and an appropriate teat ring and affords the patient with controlled sucking and swallowing without exerting a vacuum during suction. Milk flow inside the patient’s mouth can be facilitated by gently pressing on the drop-in liner. During hospitalization, both patient and parents receive feeding training from nurses. Additionally, an introduction to the orofacial regulation therapy according to the Castillo-Morales concept is provided by speech therapists [39,40,41], which the parents are encouraged to continue after discharge. In the above case, the baby was discharged four days after birth.

**Figure 11:**
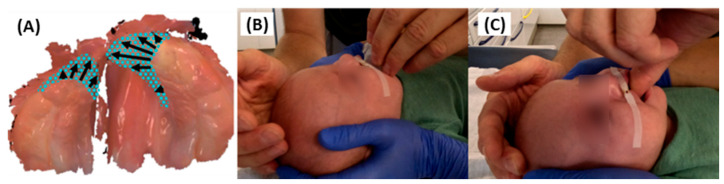
Process for the second plate in this specific case scenario involved a systematic grinding of the palatal plate (**A**). The region requiring adjustment to the presurgical palatal plate is depicted in blue, whereas the arrows refer to the expected directions for the guided growth, facilitated by the increased space between the device and the mucosa of the lateral cleft segments. The cleft covering palatal plate was then placed on the patient (age 11 weeks) by his previously trained father on the same day as the scan. The palatal plate placement in the oral cavity is illustrated (**B**), where the plate is secured in the mouth by upward pressing with a finger (**C**) [Parental informed consent was obtained to publish the information/images in an online open access publication].

Six weeks after being discharged, the patient had his first appointment at the Department of Orthodontics. At this visit, the appliance was still fitting well. Due to maxillary growth, adjustments were made to the lateral cleft segments to mimic digital wax blocking. This involved systematic grinding of the palatal plate chairside to create space for orthopedically guided growth (Figure 11). The oral cavity was examined, and no complications or side-effects were recorded. At 11 weeks of age, during the second appointment, a new appliance was indicated due to physiological growth of the infant, as the plate no longer covered the tuber region. Therefore, a new scan was obtained and a second plate produced. The IOS procedure (Figure 2), dental model (Figure 4), plate design (Figure 5) and manufacturing (Figure 6 and Figure 7) shown above were repeated for this second appliance (Figure 8). The scan was performed at 11 a.m. and the patient received the plate at 2.30 p.m. (Table 1). Parents were asked to wait, given the long journey to their residence and eliminating the need for an additional appointment. Following a brief evaluation of its oral fit by the clinician, parents were asked to insert the appliance (Figure 11) to make them feel comfortable with handling the plate after its modification. 

**Figure 12:**
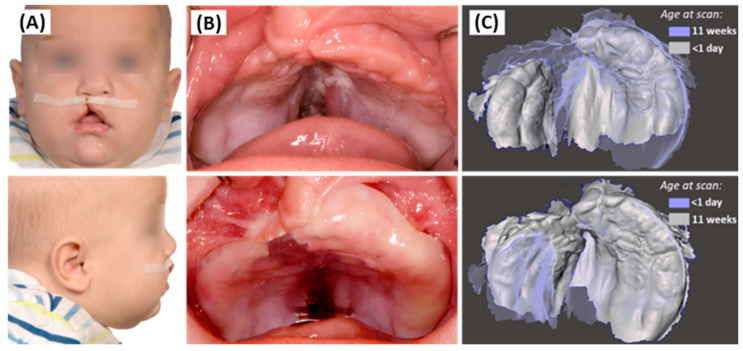
Follow-up pictures of the five-month-old boy presenting with a right-sided cleft lip and palate. (**A**) Extraoral front (**top**) and lateral (**bottom**) picture with lip tape. (**B**) Intraoral picture without (**top**) and with (**bottom**) the palatal plate appliance. (**C**) Superimposition of scans (Meshmixer software) from the same patient case at the day of birth and after 11 weeks of passive plate therapy, in two color-coded visualization types. [Parental informed consent was obtained to publish the information/image(s) in an online open access publication].

The progress and oral fit of the second palatal plate for the presented patient at age 5 months, prior to lip surgery, are shown in Figure 12. The patient did not show any adverse reactions (e.g., pressure marks) to the palatal plate treatment. The second scan demonstrated an improvement in alveolar and maxillary growth. This progress became evident between the initial condition at birth and 11 weeks later (Figure 12): Not only was the width of the cleft reduced in the palatal area but also in the alveolar ridge, achieving a harmonious alveolar shape pre-surgically.

The presented case reflects a common in-house scenario, where over 95% of patient-individualized passive palatal plates are manufactured using the described completely digital workflow (Table 3). Over a 5-year period, while bilateral cases accounted for the majority of active plates with a screw, the passive option remained prevalent for both bilateral and unilateral cases. Patients with an isolated cleft palate exclusively received passive palatal plates.

## 4. Results and Discussion

Implementing patient treatment steps in clinical practice requires careful evaluation for optimal outcomes. This workflow for treating CL/P newborns using presurgical cleft covering plates prioritizes factors from most to least important: safety, quality, user-friendliness, feasibility, and cost. Despite limited specific literature, the successful use of IOS and CAD/CAM technologies in other areas suggests their potential here.

### 4.1. Patient Data Acquisition through Intraoral Scanning

In terms of safety during the data acquisition process, the use of IOS showed superior safety in comparison to conventional alginate impressions. Alginate impressions are associated with inherent risks and complications arising from the impression material and its characteristics [26,27,28,29,30,42,43,44]. In contrast to impression taking, IOS has not been associated with any risks or complications. As a result, it can be performed without securing the airway or sedation [30,42]. In rare instances, patients may bite on the scanner head, causing minor mucosal injuries that invariably heal spontaneously [30].

Once safety considerations are assured, another crucial factor is the quality of the acquired data. In this regard, IOS surpasses conventional alginate impressions considering trueness, precision, reproducibility, and error management [43,44,45,46]. Okazaki et al. conducted an evaluation comparing conventional stone casts with models generated through IOS and a 3D printer [47]. This comparative analysis focused on models derived from the same patients with CL/P, utilizing both calliper measurements and a computer superimposition process. The study revealed significant differences between models, attributing these variations to the pressure exerted during the application of silicone impression material [47,48]. In contrast to alginate impressions, which are prone to material-induced errors, IOS precisely reproduces maxillary structures in a 1:1 ratio, capturing actual dimensions, colour, and texture reliably [30,42,48]. IOS not only provides superior trueness in representing reality, compared to conventional impressions, but also exhibits high precision and reproducibility [43,44,45,46]. Additionally, errors occur less frequently with IOS, and identifying and addressing them in the scan is easier compared to conventional methods. Here, information like colour and texture plays a major role (Figure 3) [30,42,48]. The accuracy and precision of IOS are influenced by both the scanned object and the scanning device employed [44,49]. Some studies suggest that larger scanner heads improve scan quality [43], while others have achieved suitable images using different IOS technologies for CL/P patients [38,42]. In our experience, larger dimensions of the scanner head do not hinder data acquisition. While a smaller scanner head might offer improved comfort, it can present challenges during the superimposition process for generating the 3D file. Additionally, tipping the scanner tip at the entry of the oral cavity is often sufficient for obtaining images due to the device’s depth of field [30]. The primary challenge is to capture the posterior section of the oral cavity, particularly if mouth opening is limited, such as in Robin Sequence [30]. Notably, even newborns can be successfully scanned [30]. It is important to emphasize that if a scanner cannot be used, conventional alginate impressions would also pose challenges. With advances in technology, scanner tips are expected to become more compact while maintaining high accuracy.

The existing literature lacks comprehensive coverage of intraoral scan problems and their solutions, particularly regarding newborns and young infants. Devices are typically geared towards larger patients, leading to a common oversight of specific scan requirements for patients with CLP. Consequently, both hard- and software may not meet current needs, as the anatomical structures being scanned deviate from the normal anatomy found in the vast majority of patients. Additionally, challenges arise from the lack of compliance in young patients. While errors and artefacts not addressable by scanner software are infrequent, it is essential to have an in-house protocol for handling such issues, similar to that proposed in this study. Scans from children with bilateral CLP with large premaxillary protrusion, as well as wide/deep cleft scans, constitute the most challenging scenarios. Despite this, we have only encountered these in about ¼ of cases scanned. In a multicentre examination of IOS in cleft patients, the frequency of scanning artefacts was not addressed [42]. However, the group highlighted the importance of cleaning scans to remove unnecessary image artefacts and overlapping triangles [36]. Implementing new acquisition technologies should be approached with consideration for potential errors, emphasizing the need for an alternative plan to address unforeseen issues.

After confirming the safety and quality of the process, the evaluation shifts to assessing its user-friendliness and feasibility, considering various stakeholders, such as patients, caregivers, clinicians, and the local facility. IOS demonstrates high user-friendliness and feasibility, offering benefits in patient comfort, absence of fasting requirements, and adaptability to diverse cases [50,51,52]. In terms of benefits provided to parents or guardians, as well as its acceptance [52], IOS surpasses the conventional approach; given that they can be present thorough the procedure [30] and that the scan is a more useful tool for visual representation of the patient’s anatomy [48,53,54]. However, from a facility’s perspective of implementing the data acquisition process, several disadvantages should be mentioned. This includes not only the need for a comparatively high initial investment but also a reluctance by hospital stakeholders, for the establishment of a new workflow and initial training, along with safety concerns. Additionally, maintenance demands of IOS surpass those of conventional impressions. All other aspects, however, strongly favour the implementation of the scanning approach, particularly if it comes to human resource requirements and preparatory steps [30,42]. Unlike conventional impressions that necessitate a complete team for anaesthesia and emergency situations, IOS can be performed chairside by a single clinician in the presence of a parent. Additionally, in terms of organizational issues and prior preparation, the conventional process is more complex to coordinate, considering the availability of professionals and equipment for both, as well as preparation for the alginate impressions (e.g., individual impression trays). As these are unnecessary for IOS, the procedure inherently carries a lower burden of organization and preparation, resulting in an overall reduction in data acquisition time [30,42]. Given the unique requirements of the conventional impression, specific facility rooms with specialized equipment are essential for the appointment, whereas IOS can be conveniently performed chairside. Apart from this, IOS also offers more processing, storage, backup, data control, and data transfer options than conventional impression-taking, which typically requires an intermediary step of digitalization through an extraoral scanner. Moreover, successful data acquisition is less dependent on intrinsic characteristics with IOS than with alginate impressions. Different authors agree with the current study that the implementation of a digital acquisition technique requires less personnel while increasing efficiency [37,42].

Once all other aspects are assured, the process should be planned to achieve highest efficiency at lowest possible expense. Solely considering routine material use, excluding infrastructure, initial investment, and necessary human resources, IOS surpasses the conventional procedure in both time and cost requirements (Table 4). Zarean et al. showed similar results for intraoral scanning time, reporting an exemplary 15 min compared to our facility’s 7 min [36]. They reported 1 h and 10 min for the conventional impression-taking procedure [36]. Despite both values being higher than those presented in our study, Zarean et al. did not specify details of this estimation. It is believed that a margin of error was included for both scenarios.

Thus, IOS technology outperforms conventional impression-taking in various aspects when considering newborns and young infants with clefts. Overcoming challenges related to initial investment or costs and integrating it into a routine protocol could result in completely replacing conventional impressions in clinical practice for this clientele. 

### 4.2. Use of CAD/CAM for the Creation and Manufacturing of the Presurgical Cleft Covering Palatal Plate

Concerning the safety level in the design and manufacturing of these appliances, both processes offer similar safety to patients, clinicians, and technicians, but with a slight advantage favouring digital technologies for the latter. Exposure to materials and alcohol can be expected in both processes, which needs to be considered in the safety routine of the technician. However, considering less hands-on time and tool usage associated with CAD/CAM technologies, they are deemed marginally safer. Additionally, given that employed materials meet MDR guidelines and are manufactured accordingly, both processes prove to be safe for handling by parents and for their use in patients.

Regarding the quality level of the two different design and manufacturing approaches, CAD/CAM technologies are anticipated to yield higher trueness, precision, and reproducibility [31]. This is attributed to the intrinsic feature of reduced hands-on time in CAD/CAM approaches. Although a certain margin of error can also be expected, this is not quantifiable with existing technologies, with this error being the sum of the different steps within the digital workflow, making their identification inviable [31]. A previous study evaluated CAM technologies for manufacturing palatal plates, demonstrating that subtractive manufacturing (milling) and DLP manufacturing technologies comprised both, significantly higher trueness and precision to the conventional approach, whereas SLA showed significantly decreased trueness, but increased precision [31]. Milling demonstrated superior trueness and accuracy than additive manufacturing (AM), while digital light processing (DLP) offered higher trueness and precision to stereolithography (SLA) [31]. Despite variations between manufacturers and materials, the study confirmed that CAM provides comparable, if not superior, trueness and precision to conventional manufacturing for palatal plates [31]. In the context of this application, both trueness and precision stand as crucial accuracy parameters. Trueness is defined as the agreement between the expected measurement result and the true value. It is vital for the fitting of the palatal plate, ensuring it follows the real information obtained from the maxilla. Precision is essential for consistent quality output, ensuring that each appliance is manufactured with the same level of accuracy [31]. Palatal plates, susceptible to loss or breakage by the caregivers, can be precisely reproduced in case of damage, maintaining identical quality to the original. While the expertise of a dental technician is significant in conventional dental lab techniques, CAD/CAM technologies can aid in reducing potential human errors by minimizing hands-on steps in creating patient-specific appliances [31,35].

Once safety and quality are assured in the design and manufacturing processes, the evaluation extends to user-friendliness and feasibility. In terms of patient cases, CAD/CAM technologies prove versatile by addressing a broad range of scenarios without reported limitations for passive palatal plates. Following established minimum quality standards for scans, no design or manufacturing constraints are expected. For dental technicians, a digital approach offers greater comfort due to reduced hands-on time (Table 4). Nevertheless, it demands a higher learning curve and expertise, necessitating proficiency in basic CAD knowledge, familiarity with preferred CAM technologies, and manual dental laboratory steps. Additionally, the plate can be directly manufactured without the need for prior model manufacturing. If desired, the model can be manufactured using a wide range of AM machines and materials. This can be useful for describing the information of the therapy to caregivers, as well as control its fit in vitro. Regarding software selection, the current study employed a license based dental software, whereas other studies use freeware solutions for designing the palatal plate [36,37], particularly Meshmixer, which was previously introduced for scan repair. Both software options proved to be feasible, although the licensed and commercially available dental software typically provides a more user-friendly environment as well as a medically approved product. It requires less training and no prior CAD experience. Additionally, it enables faster and easier addressing of errors, while freeware demands a higher expertise. Furthermore, the dental appliance designer and dental manager software are directly connected to the scanner software. This ensures smooth information retrieval within the facility network, without any breach in patient security or mixing up patient files. Despite the higher entry and maintenance costs associated with implementing CAD/CAM technologies, it reduces the demand for human resources and enables multiple simultaneous manufacturing processes. Digitalization for storage, data exchange, and backups is inherent to the digital process without the need for an additional step of extraoral scanning. Each design and manufacturing file, saved with respective information, enables retracing the process to address potential pitfalls or retrospective improvements—an aspect hardly attainable with conventional methods.

Table 5 provides approximate times for appliance fabrication, which can vary depending on the individual patient. The total fabrication time is estimated at approximately 2 h and 25 min for the conventional method, while the digital method takes 20 min less. Crucially, the digital fabrication time does not involve hands-on time for the technician, as it is independently performed by the DLP machine. Other studies have employed different CAM technologies for plate production, such as SLA [36] and subtractive manufacturing [31,32]. In a publication considering a complete digital workflow for passive palatal plate manufacturing, the authors estimated a design time of 35 min, which matches the 15 min for model fabrication and 20 min for plate design in our workflow [36]. Additionally, they reported a manufacturing time of 90–180 min (dependent on the printed volume), AM post-processing of 110 min, and conventional post-processing of 10 min, using SLA technology [36]. Both values for the AM technology are higher than those reported here using DLP, while the conventional post-processing time is the same. 

While timing is related to the employed device and material, printing time using DLP machines is not dependent on the volume or quantity of parts. In contrast, for SLA and subtractive manufacturing, time scales are proportional to volume, hence increasing linearly with more samples [31]. Given that DLP is not dependant on the printed volume, it includes the capability to produce more than one appliance for the same patient or different appliances for various patients concurrently (Figure 6). Consequently, only manual or conventional post-processing (Figure 7) and material costs need to be accounted for each additional appliance, resulting in an extra cost of €25.77 per plate—significantly less than the conventional method. This is particularly valuable considering the high rate of plate loss by caregivers, making a backup plate highly recommended. Simultaneous manufacturing of such backup plates can save human resources, time, and costs. While manufacturing efficiency is closely related to the facility’s resources, investing in a DLP machine is considered more appropriate for enhancing manufacturing efficiency [35]. Excluding initial infrastructure investments, such as the scanner, software license, and printer, both workflow time and expenses are lower for the digital approach (Table 5). We are unaware of other cost estimations from complete palatal plate digital workflows from the literature, limiting comparability with other centres.

Establishing an in-house workflow that aligns with the above criteria enables cleft centres to reduce the interval between data acquisition and the delivery of medical appliances to patients, as demonstrated in this study’s case scenario. Patients can receive the appliance within a few hours after scanning, a feat not achievable through outsourcing due to extended production and delivery times, alongside increased costs. These elevated costs diminish the facilities’ margins, potentially rendering the treatment economically unviable. However, an in-house, fully digital workflow, enables a reduction of both costs and delivery times. Additionally, it facilitates communication between dental technicians and clinicians, potentially improving treatment outcomes.

Moreover, considering the global trends towards digitalization and automation, alongside a declining interest in hands-on or conventional professions, the consideration of digitalizing workflows becomes imperative for ensuring sufficient human resources in cleft centres. Minimizing required training and hands-on time makes it possible for individuals with diverse training backgrounds from interdisciplinary teams—including clinicians (orthodontists, surgeons, dentists, neonatologists), assistants, and researchers—to potentially participate in the workflow.

### 4.3. Treatment Outcome

The presented patient case demonstrates a notable enhancement in maxillary anatomy after passive palatal plate therapy, achieving a physiological shape that not only reduces the cleft defect on the alveolar ridge but also addresses the palate, as well as achieving a more harmonic dental arch. Other studies employing a passive plate approach reported similar outcomes [55]. The successful realization of this outcome hinges on the meticulous execution of fundamental steps, including digital wax blocking of the model and adherence to clinical control appointments. During these appointments, it is imperative to create appropriate space on the appliance and to implement correct lip taping to guide growth effectively [22]. 

Apart from the discredited theory proposed by McNeil and Scott [4,5], various theories regarding the function of PSIO have been debated. One widely accepted perspective is the “Functional Matrix Principle” suggested by Moss [56,57]. According to this principle, bone and cartilage growth relies on the soft tissue, with their interaction regulated by their functional relationship. The functional soft tissue matrix is proposed as the primary influencer of skeletal growth, impacting the response of hard tissue reciprocally. This theory contends that skeletal growth depends on primary changes in the functional matrix. The use of a passive palatal plate serves to mitigate negative growth influences, such as tongue pressure, and to facilitate physiological growth in the region where it is needed. Additionally, it enables the establishment of a natural tongue position, contributing to a harmonious swallowing pattern. The latter fosters a physiological drinking behaviour, crucial for weight gain and age-appropriate development in the initial weeks of life.

Despite the fact that many studies support presurgical cleft alignment through PSIO [7,12,58,59,60], the use of presurgical orthodontic appliances for the treatment of CL/P remains controversial [2,61]. The “Dutchcleft Study” [62,63] assessed orthodontic results using plaster cast models measured three-dimensionally at different ages. The results indicated a temporary increase in maxillary arch dimension before surgical closure of the lip. However, at the age of 1½ years after soft palate closure, dimensions decreased again, resembling the outcomes of patients that did not receive presurgical treatment. Long-term results at age 6 years suggested a similar pattern, with collapse of the cleft segments and persistence of malocclusive situations [64]. Only the speech outcome at age of 2–2 ½ years was found to be improved in the treated group [65]. The study showed that holding and stabilizing the aligned segments after lip closure and primary bone grafting surgery was the main problem. This arises from tension exerted by scar tissue and the early surgical intervention, which inhibits growth [2,66,67,68]. Rosenstein recommended early presurgical orthopaedics, followed by later orthodontic treatment to address functional conditions [8]. The long-term impact on maxillofacial growth and speech development was considered as the primary outcome in PSIO. Thus, it seems reasonable to assume that a carefully chosen surgical timepoint could also yield benefits [3,69]. Considering this, the interdisciplinary treatment concept described here becomes even more important for a successful rehabilitation. In cases where the cleft defect is too large for surgery, resulting in large forces and scarring, the benefits of this type of presurgical treatment are most pronounced. Additionally, in large bilateral cleft defects with an extremely protruding premaxilla and no space between both lateral alveolar segments, presurgical treatment might be particularly advantageous. This approach allows for an initial transversal expansion of the maxilla and facilitates the posterior premaxilla alignment into the dental arch for later surgery.

Unfortunately, to date, there is an absence of long-term follow-up data, primarily attributable to the absence of standardized protocols for cleft care. This was confirmed in 2022 through a survey involving 115 international cleft teams, most of which (n = 78) employed PSIO, despite conflicting evidence [70]. Among these, 65 centres implemented alveolar moulding, with only 10 adapting a passive plate concept. Lip taping in combination with plate therapy was administered by 45 teams. Furthermore, the initiation of therapy typically occurred after one week of age, later than with the Tübingen approach. While most teams involved various medical disciplines, PSIO was predominantly provided by orthodontists [70].

In Europe, 53% of cleft centres incorporate PSIO into their treatment approaches [71]. The wide spectrum of therapy concepts stems from the low prevalence of individuals with CL/P, coupled with the complex and variable anatomical conditions. Consequently, the resultant small sample size available for study designs leads to considerable heterogeneity, hindering meaningful comparisons. This scenario emphasizes the dearth of conclusive evidence guiding the international use of neonatal CL/P care. Despite the inclination of expert and centre towards employing PSIO, the current state of evidence poses challenges in definitively justifying the adoption of this approach.

### 4.4. Limitations

As previously mentioned, the potential adoption of a digital workflow is closely related to each centre’s resources, including IOS soft- and hardware. The present study does not account for initial investment costs for both conventional and digital approaches, as these vary widely between countries, manufacturers, and market conditions. The authors aim to avoid bias towards specific solutions or machines, instead focusing on demonstrating feasibility and operational efficiency. This approach allows institutions to use existing resources or consider new investments based on their specific contexts. Additionally, the provided information may quickly become outdated as technological advances and market competition drives costs down, increasing accessibility over time. 

Considering time estimations, these were taken from the master file employed to calculate expected costs for insurance cost for each patient, defined by the potential variability in each case. Moreover, the initial duration required to implement the workflow into clinical routine and to train all involved parties has not been considered. This requires not only time but also the acceptance and collaboration of the entire team, including employees dealing with related paperwork (e.g., treatment costs, insurance, documentation systems, MDR documentation). 

Although the described workflow focuses exclusively on passive palatal plates, this was found to provide a solution for up to 95% of devices needed in-house (Table 3), as passive plates were predominantly used in cleft care. Meanwhile, active palatal plates, e.g., plates with orthodontic expansion screws, could be produced following a semi-digital workflow. This involves producing the IOS-based and AM-manufactured dental model from which a conventionally manufactured palatal plate could be produced. Other active palatal plate approaches, such as NAM, are not considered by our team. This is due not only to conflicting literature but also to an increased burden of care, rendering it a less attractive solution [72]. Furthermore, the fabrication of NAM devices is currently only achievable through semi-digital workflows [73,74,75,76,77,78], as the existing CAD/CAM technologies, at present, face limitations, particularly in establishing a connection between the steel wire of the extraoral device and the polymer of the NAM plate. This specific manufacturing step must be carried out conventionally in dental labs, as digital methods are not yet available [79]. Consequently, the use of a complete digital workflow is currently confined to passive palatal plate manufacturing. 

Furthermore, this manuscript focuses on presenting a methodology while providing a case scenario for comprehension purposes based on a single scenario. As indicated in Table 3, only 23% of cleft patients have a bilateral CLP. Typically, these cases involve a wide cleft on one side and an incomplete cleft on the other, with prominent premaxillae representing the least common occurrence, approximately a quarter of all bilateral cases. Given that the design process for CL/P passive plates differs while the remaining information (e.g., treatment, manufacturing process) remains identical, repeating much of the given information would be redundant. Therefore, this approach was not detailed. However, it is important to emphasize that cases with a prominent premaxilla can be particularly challenging, and the workflow presented here may not be applicable in every scenario. Due to the multifaceted nature of treatment for such cases, we could not encompass this within the publication due to length constraints.

## 5. Conclusions

Within the limitations of the current study, and considering the already existing infrastructure, the following conclusions can be drawn from this report:

1. IOS demonstrated superiority over conventional impressions in terms of safety, quality, user-friendliness, feasibility, and cost-effectiveness.
2. The use of a CAD/CAM protocol, when compared to conventional impressions, was found to be safer, higher in quality, more user-friendly, feasible, and cost-effective.
3. In the presented exemplary case, presurgical passive palatal plate treatment showed significant progress by reducing the cleft width and promoting enhanced physiological arch formation within an 11-week period.

Despite the controversy surrounding the use of PSIO, streamlining the process and minimizing drawbacks associated with traditional methods can enhance its implementation in facilities worldwide. Simultaneously, ensuring cost-effectiveness may facilitate its coverage by insurance companies.

While digital orthodontic care for neonates is in its infancy, it holds considerable potential that can be expected to further improve with technological development. Additionally, digital documentation in healthcare provides an opportunity to leverage advanced technologies, offering not only solutions but also feedback to enhance treatment strategies. Neonates and young infants with CL/P often require complex orthodontic treatment, involving specialized techniques like PSIO. Integrating digitalized PSIO facilitates achieving anatomically accurate cleft conditions. Considering individual anatomy and understanding facial growth patterns before treatment initiation is crucial.

Further studies can assess CL/P maxilla anatomy using IOS and standardized measurements, providing valuable insights into long-term development and growth predictions. This information lays the foundation for incorporating machine learning in cleft care. Furthermore, digitalization enables telehealth medicine, simplifying collaboration, education, and remote patient support, ensuring a heightened level of care.

## Figures and Tables

**Table 1:** Timeline of the performed digital workflow in this exemplary case, where in each appointment, following intraoral scanning, a new personalized palatal plate was manufactured.

Case Scenario	1st Palatal Plate	2nd Palatal Plate
Age	<1 day	11 weeks
**Scan**		
Appointment time	9 a.m.	11 a.m.
Preparation scanner	about 5 min	about 5 min
Scanning procedure		
→ Time	3 min 16 s	1 min 44 s
→ Number of pictures	1257	1480
Post-processing scan	N/A	N/A
**CAD**		
Digital model	10 min	
Cleft covering palatal plate	15 min	
**CAM**		
Slicing + printing preparation	Ca. 10 min	
Manufacturing in machine		
→ Duration	38 min 15 s	48 min 19 s
→ Number of layers (layer thickness = 100 µm)	256	320
**Post-processing**		
Post-processing AM material	25 min	
Dental lab post-processing	about 10 min	
Delivery time (hh:mm)	4 p.m.	2.30 p.m.
Total waiting time patient	7 h	3 h 30 min

**Table 2:** Parameters to evaluate during each orthodontic appointment for palatal plate fitting include extent, rotation, overturning, mucosal peculiarities, and other factors. Clinical pictures for each parameter and the corresponding therapy steps are provided.

Palatal Plate Evaluation		Clinical Picture	Course of Action
**Extent**	*Posterior*	Cover the maxillary tuber region and end at the soft palate to ensure unhindered movement, such as during swallowing, and to avoid triggering the gag reflex.	If the base of the palatal plate is too short, indication for a new IOS If it is too long, grinding and polishing at the appropriate point
	*Lateral*	Extend as widely as possible in the muco-buccal fold at the attached gingiva, while relieving the lateral frenulum to allow for its movement.	
	*Anterior* *(cleft region)*	Relieve the vestibular region to provide adequate space for lip taping and surgical lip repair. Adjustments to the gained space in this region will be necessary during each follow-up visit.	
**Rotation** **(horizontal dimension)**		*Cause:* If the appliance becomes too small, it may rotate horizontally on the maxilla as its rim loses grip in the muco-buccal fold. *Recurrence:* Arises always due to physiological growth.	New IOS is indicated
**Tilting** **(vertical dimension)**		*Cause:* Incorrect IOS or misalignment of cleft segments (wide/deep cleft conditions). *Recurrence:* Occurs only if the appliance does not fit the anatomy.	New IOS is indicated
**Mucosal local** **peculiarities**	*Pressure marks* (*traumatic* *ulcerations*)	*Appearance:* oval shaped white spot with a red edge and the size of a lentil. *Recurrence:* rare. *Pain:* yes, must be addressed before resuming treatment.	Plate needs to be adjusted chairside (grinding, polishing) to provide space in between the plate and the mucosa for the healing process. In certain cases, new IOS is necessary.
	*Indentations*	*Appearance:* Impressions with an intact mucosal surface. *Recurrence:* Arises always if appliance is too small or malpositioned. *Pain:* none, tolerable during treatment.	In the case of a new device: Virtual wax blocking in this region, that the resilient mucosa can normalize.
**Other** **considerations**	*Neonatal teeth*	*Recurrence:* Rare, mostly in the cleft region or mandibular front region.	Surgical extraction needed
	*Odontiasis of a* *deciduous tooth*	*Recurrence:* always as the treatment progresses	In the anterior region: plate therapy still possible by removal of material according to affected region (grinding, polishing) In the posterior region: therapy must be finished
	*Mucosal* *general* *peculiarities*, e.g., *thrush*	*Recurrence:* rare	Increasing the oral hygiene, e.g., frequent plate changes and cleaning

**Table 3:** Intraoral scans done between 10/2018 and 10/2023. All scans led to the creation of an individualized palatal plate, comprising passive plates through a complete digital workflow and active plates via a semi-digital process (IOS-based AM model fabrication for conventional plates with screws). Scans were categorized into unilateral (UCL/P) and bilateral cleft lip and/or palate (BCL/P), as well as into isolated cleft palate (CP) cases without diagnosis of a syndrome or Robin sequence. Results are shown as counts (n) and percentages (%) for each group and the total sample.

Count (n)	Scans	Passive/Active Palatal Plate Ratio	Patients	
UCL/P	255 (68.92%)	245/10 (96.08/3.92%)	96 (97.61%)	Total: 96 (100%)
				Right CL/P: 31 (32.29%)
				Right CL: 1 (1.04%)
				Left CLP: 59 (61.46%)
				Left CL: 5 (5.21%)
BCL/P	85 (22.97%)	68/17 (80/20%)	32 (22.54%)	
CP	30 (8.11%)	30/0 (100/0%)	14 (9.86%)	
Total	370 (100%)	343/27 (92.70/7.30%)	142 (100%)	

**Table 4:** Evaluation of aspects concerning time efficiency and expense level for data acquisition for newborns and infants with CL/P, comparing conventional alginate impression to intraoral scanning (IOS). IOS times are based on a previous study [30]. The outcome defines the level of efficiency and expense provided by each of the approaches. The total cost does not consider infrastructure and initial investment costs. “>” or “<” marks which approach requires more time/costs.

Efficiency and Expense Level—Data Acquisition			
	Alginate Impression	>/<	IOS
Preparing setting for impression taking	15 min		5 min
Individual impression tray manufacturing	15 min		N/A
Informing neonatal team, fixation extra appointment	5 min		N/A
Impression taking process	5 min		2 min
**Total time**	40 min	**>**	7 min
Individual impression tray	65.00 €		N/A
Impression material	3.00 €		N/A
**Total costs**	68.00 €	**>**	N/A

**Table 5:** Evaluation of aspects concerning the efficiency and expense level for appliance design and manufacturing for newborns and infants with CL/P, either by the conventional dental lab or CAD/CAM technologies. Total cost does not consider infrastructure and initial investment costs but considers personnel hands-on time and associated hour work rate price for dental technician. (*) denotes hands-on time. “>” or “<” marks which approach requires more time/costs.

Efficiency and Expense Level—Appliance Design and Manufacturing			
	Conventional Dental Lab	>/<	CAD/CAM Technologies
Model fabrication	30 min (* 15 min)		(*) 15 min (virtual design)
Palatal plate design	N/A		(*) 20 min
Fabrication time	(*) 1 h 10 min		1 h 3 min
Post-processing AM material	N/A		(*) 17 min
Post-processing conventional	(*) 45 min		(*) 10 min
Total hands-on (*) time	**2 h 10 min**	**>**	**1 h 2 min**
Model fabrication	7.00 €		25.00 €
Palatal plate design	N/A		50.00 €
Palatal plate material	0.24 €		0.77 €
Fabrication	65.00 €		17.00 €
Post-processing AM material	N/A		33.00 €
Post-processing conventional	20.00 €		25.00 €
**Total fabrication costs**	**Approx. 92.24 €**	**<**	**Approx. 151.00 €**
**Total workflow time**	**2 h 25 min**	**>**	**2 h 5 min**
**Total workflow costs** **(fabrication + data acquisition)**	**Approx. 160.24 €**	**>**	**Approx. 151.00 €**

## Data Availability

The datasets generated and/or analysed during the current study are not publicly available due patient data protection. Additional information about the methodology and implementation is available upon reasonable request to the corresponding author.
